# A Neglected Disinfectant

**Published:** 1883-12

**Authors:** 


### A NEGLECTED DISINFECTANT.

When the household of our grandmothers was threatened with infection the common practice was to sprinkle brimstone on a hot shovel or on hot coals on a shovel, and carry the burning result through the house. But now this simple method of disinfecting has gone out of fashion without any good and sufficient reason.

The principal reason is neither good nor sufficient, viz., that nobody can patent it and sell it in twenty-five and fifty-cent bottles. On the 18th of September last M. d'Abbadie read a paper at the French Academy on "Marsh Fevers," and stated that in the dangerous regions of African river mouths immunity from such fevers is often secured by sulphur fumigations on the naked body. Also that the Sicilian workers in low-ground sulphur mines suffer much less than the rest of the surrounding population from intermittent fevers. M. Fouqué has shown that Zephyria (on the volcanic island of Milo or Melos, the most westerly of the Cyclades), which had a population of 40,000 when it was the centre of sulphur mining operations, became nearly depopulated by marsh fever when the sulphur mining was moved further east and the emanations prevented by a mountain from reaching the town. Other similar cases were stated.

